# Crystal structure of fenbuconazole

**DOI:** 10.1107/S205698901501542X

**Published:** 2015-08-22

**Authors:** Gihaeng Kang, Jineun Kim, Hyunjin Park, Tae Ho Kim

**Affiliations:** a Department of Chemistry and Research Institute of Natural Sciences, Gyeongsang, National University, Jinju 52828, Republic of Korea

**Keywords:** crystal structure, fungicide, fenbuconazole, C—Cl⋯π inter­actions, π–π inter­actions

## Abstract

In the title compound, C_19_H_17_ClN_4_ [systematic name: (*RS*)-4-(4-chloro­phen­yl)-2-phenyl-2-(1*H*-1,2,4-triazol-1-ylmeth­yl)butyro­nitrile], which is the conazole fungicide fenbuconazole, the dihedral angles between the planes of the central benzene and the terminal chloro­phenyl and triazole rings are 32.77 (5) and 32.97 (5)°, respectively. The C—C—C—C linkage between the tertiary C atom and the benzene ring has an *anti* orientation [torsion angle = 174.47 (12)°]. In the crystal, C—H⋯N hydrogen bonds and very weak C—Cl⋯π inter­actions [Cl⋯π = 3.7892 (9) Å] link adjacent mol­ecules, forming two-dimensional networks lying parellel to the (101) plane. The planes are linked by weak π–π inter­actions [centroid–centroid separation = 3.8597 (9) Å], resulting in a three-dimensional architecture.

## Related literature   

For information on the fungicidal properties of the title compound, see: Li *et al.* (2012 ▸). For related crystal structures, see: Rizzoli *et al.* (2009 ▸); Yin *et al.* (2014 ▸).
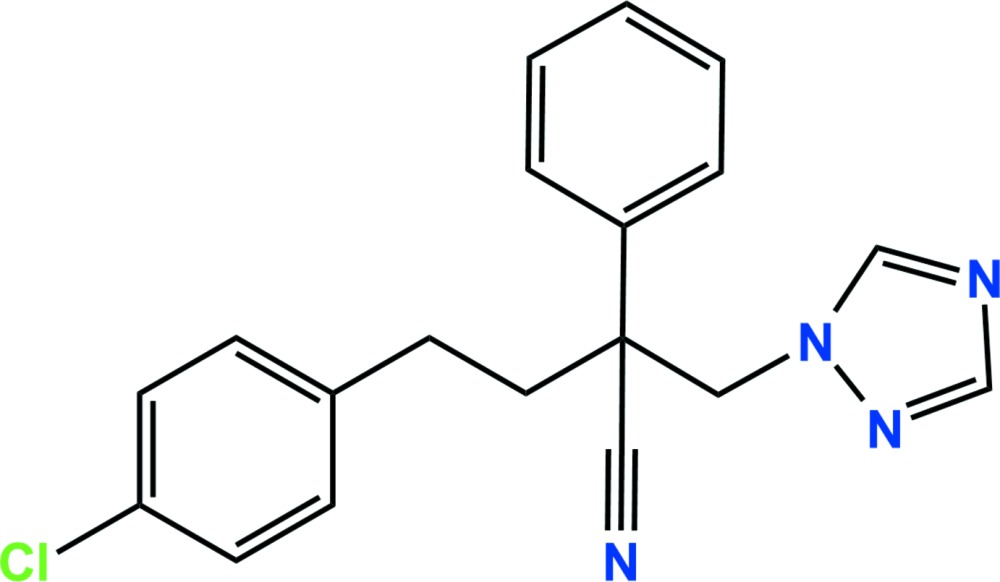


## Experimental   

### Crystal data  

C_19_H_17_ClN_4_
*M* *_r_* = 336.82
Monoclinic, 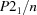

*a* = 12.4606 (3) Å
*b* = 6.7404 (2) Å
*c* = 20.5394 (5) Å
β = 95.455 (2)°
*V* = 1717.28 (8) Å^3^
*Z* = 4
Mo *K*α radiation
μ = 0.23 mm^−1^
*T* = 173 K
0.18 × 0.07 × 0.03 mm

### Data collection  

Bruker APEXII CCD diffractometer
Absorption correction: multi-scan (*SADABS*; Bruker, 2013 ▸) *T*_min_ = 0.959, *T*_max_ = 0.993
15784 measured reflections
3936 independent reflections
3044 reflections with *I* > 2σ(*I*)
*R*_int_ = 0.035

### Refinement  

*R*[*F* ^2^ > 2σ(*F* ^2^)] = 0.044
*wR*(*F* ^2^) = 0.108
*S* = 1.04
3936 reflections
217 parameters
H-atom parameters constrained
Δρ_max_ = 0.25 e Å^−3^
Δρ_min_ = −0.30 e Å^−3^

### 

Data collection: *APEX2* (Bruker, 2013 ▸); cell refinement: *SAINT* (Bruker, 2013 ▸); data reduction: *SAINT*; program(s) used to solve structure: *SHELXS97* (Sheldrick, 2008 ▸); program(s) used to refine structure: *SHELXL2013* (Sheldrick, 2015 ▸); molecular graphics: *DIAMOND* (Brandenburg, 2010 ▸); software used to prepare material for publication: *SHELXTL* (Sheldrick, 2008 ▸).

## Supplementary Material

Crystal structure: contains datablock(s) global, I. DOI: 10.1107/S205698901501542X/hb7481sup1.cif

Structure factors: contains datablock(s) I. DOI: 10.1107/S205698901501542X/hb7481Isup2.hkl

Supporting information file. DOI: 10.1107/S205698901501542X/hb7481Isup3.cml

**Fig. 1.**
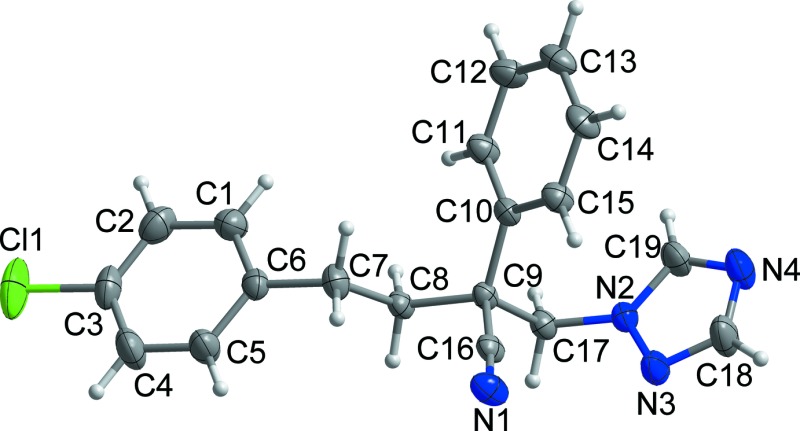
The asymmetric unit of the title compound with displacement ellipsoids drawn at the 50% probability level.

**Fig. 2.**
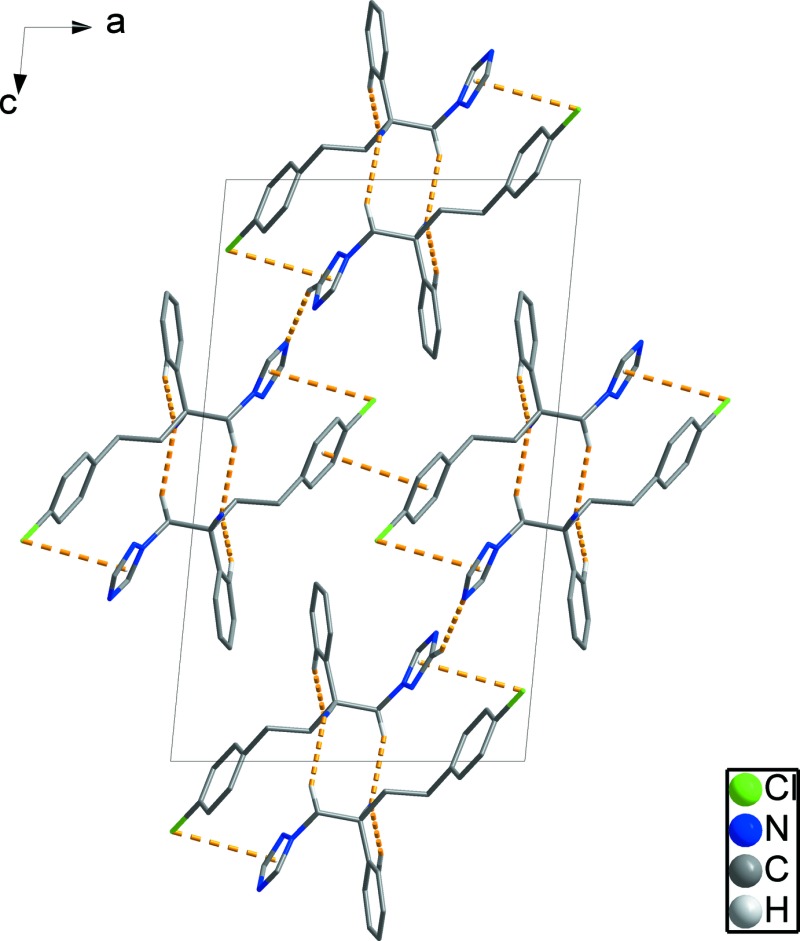
Crystal packing viewed along the b axis. The intermolecular interactions are shown as dashed lines.

. DOI: 10.1107/S205698901501542X/hb7481fig1.tif
The asymmetric unit of the title compound with displacement ellipsoids drawn at the 50% probability level.

b . DOI: 10.1107/S205698901501542X/hb7481fig2.tif
Crystal packing viewed along the *b* axis. The inter­molecular inter­actions are shown as dashed lines.

CCDC reference: 1419334

Additional supporting information:  crystallographic information; 3D view; checkCIF report

## Figures and Tables

**Table 1:** Hydrogen-bond geometry (, )

*D*H*A*	*D*H	H*A*	*D* *A*	*D*H*A*
C8H8*A*N1^i^	0.99	2.53	3.522(2)	178
C11H11N1^i^	0.95	2.60	3.533(2)	166
C17H17*A*N1^ii^	0.99	2.58	3.5101(18)	156
C18H18N4^iii^	0.95	2.46	3.277(2)	144

Symmetry codes: (i) 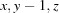; (ii) 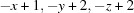; (iii) 
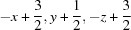
.

## supplementary crystallographic information

### S1. Comment

Fenbuconazole, [systematic name: (*RS*)-4-(4-chlorophenyl)-2-phenyl-2-(1*H*-1,2,4-triazol-1-ylmethyl)butyronitrile], is a conazole fungicide and it has been used for the control of leaf spot, yellow and brown rust, powdery mildew, and net blotch on various agricultural and horticultural crops (Li *et al.*, 2012). However, until now its crystal structure has not been reported. The dihedral angles between the planes of the central benzene and the terminal chlorophenyl and triazole rings are 32.77 (5) and 32.97 (5)°, respectively. All bond lengths and bond angles are normal and comparable to those observed in similar crystal structures (Rizzoli *et al.*, 2009; Yin *et al.*, 2014).

In the crystal structure (Fig. 2), C—H··· N hydrogen bonds and weak C3–Cl1···*Cg*1^iv^ (*Cg*1 is the centroid of the N2–N3–C18–N4–C19 ring) interaction [3.7892 (9) Å] with a chlorophenyl ring are observed (Table 1), forming two-dimensional networks parelle to (101) plane. In addition, the planes are linked by weak intermolecular π···π interaction between the terminal chlorophenyl ring systems [*Cg*2···*Cg*2^v^, 3.8597 (9) Å], resulting in a three-dimensional architecture. (*Cg*2 is the centroid of the C1–C6 ring) [for symmetry codes: (iv), -*x* + 1, -*y* + 1, -*z* + 2, (v), -*x*, -*y* + 1, -*z* + 2].

### S2. Experimental

The title compound was purchased from the Dr Ehrenstorfer GmbH Company. Slow evaporation of a solution in CH_3_CN gave brown plates suitable for X-ray analysis.

### S3. Refinement

All H-atoms were positioned geometrically and refined using a riding model with d(C—H) = 0.99 Å, *U*_iso_ = 1.2*U*_eq_(C) for CH_2_ group and d(C—H) = 0.95 Å, *U*_iso_ = 1.2*U*_eq_(C) for aromatic C—H.

### Crystal data

**Table:**

C_19_H_17_ClN_4_	*F*(000) = 704
*M**_r_* = 336.82	*D*_x_ = 1.303 Mg m^−^^3^
Monoclinic, *P*2_1_/*n*	Mo *K*α radiation, λ = 0.71073 Å
*a* = 12.4606 (3) Å	Cell parameters from 3787 reflections
*b* = 6.7404 (2) Å	θ = 3.2–27.4°
*c* = 20.5394 (5) Å	µ = 0.23 mm^−^^1^
β = 95.455 (2)°	*T* = 173 K
*V* = 1717.28 (8) Å^3^	Plate, brown
*Z* = 4	0.18 × 0.07 × 0.03 mm

### Data collection

**Table:**

Bruker APEXII CCD diffractometer	3044 reflections with *I* > 2σ(*I*)
φ and ω scans	*R*_int_ = 0.035
Absorption correction: multi-scan (*SADABS*; Bruker, 2013)	θ_max_ = 27.5°, θ_min_ = 1.8°
*T*_min_ = 0.959, *T*_max_ = 0.993	*h* = −15→16
15784 measured reflections	*k* = −8→8
3936 independent reflections	*l* = −26→26

### Refinement

**Table:**

Refinement on *F*^2^	0 restraints
Least-squares matrix: full	Hydrogen site location: inferred from neighbouring sites
*R*[*F*^2^ > 2σ(*F*^2^)] = 0.044	H-atom parameters constrained
*wR*(*F*^2^) = 0.108	*w* = 1/[σ^2^(*F*_o_^2^) + (0.043*P*)^2^ + 0.4967*P*] where *P* = (*F*_o_^2^ + 2*F*_c_^2^)/3
*S* = 1.04	(Δ/σ)_max_ = 0.001
3936 reflections	Δρ_max_ = 0.25 e Å^−^^3^
217 parameters	Δρ_min_ = −0.30 e Å^−^^3^

### Special details

**Table:**

Geometry. All e.s.d.'s (except the e.s.d. in the dihedral angle between two l.s. planes) are estimated using the full covariance matrix. The cell e.s.d.'s are taken into account individually in the estimation of e.s.d.'s in distances, angles and torsion angles; correlations between e.s.d.'s in cell parameters are only used when they are defined by crystal symmetry. An approximate (isotropic) treatment of cell e.s.d.'s is used for estimating e.s.d.'s involving l.s. planes.

### Fractional atomic coordinates and isotropic or equivalent isotropic displacement parameters (Å^2^)

**Table:**

	*x*	*y*	*z*	*U*_iso_*/*U*_eq_	
Cl1	0.02411 (4)	0.19334 (10)	1.12090 (3)	0.0689 (2)	
N1	0.42210 (11)	1.1422 (2)	0.92410 (6)	0.0367 (3)	
N2	0.63664 (9)	0.7989 (2)	0.86689 (5)	0.0277 (3)	
N3	0.66105 (11)	0.9948 (2)	0.87314 (6)	0.0398 (3)	
N4	0.71532 (12)	0.8721 (2)	0.77946 (7)	0.0445 (4)	
C1	0.17939 (13)	0.3822 (3)	0.97206 (8)	0.0384 (4)	
H1	0.2005	0.3286	0.9325	0.046*	
C2	0.12369 (14)	0.2639 (3)	1.01229 (9)	0.0447 (4)	
H2	0.1056	0.1311	1.0003	0.054*	
C3	0.09501 (12)	0.3415 (3)	1.06994 (8)	0.0410 (4)	
C4	0.12079 (14)	0.5320 (3)	1.08810 (8)	0.0485 (5)	
H4	0.1014	0.5833	1.1284	0.058*	
C5	0.17565 (13)	0.6493 (3)	1.04672 (8)	0.0398 (4)	
H5	0.1933	0.7822	1.0589	0.048*	
C6	0.20501 (10)	0.5763 (2)	0.98807 (7)	0.0278 (3)	
C7	0.26520 (11)	0.7035 (3)	0.94298 (7)	0.0316 (3)	
H7A	0.2559	0.8451	0.9541	0.038*	
H7B	0.2341	0.6831	0.8973	0.038*	
C8	0.38533 (11)	0.6541 (2)	0.94830 (6)	0.0237 (3)	
H8A	0.3937	0.5096	0.9418	0.028*	
H8B	0.4171	0.6864	0.9931	0.028*	
C9	0.44939 (10)	0.7655 (2)	0.89855 (6)	0.0218 (3)	
C10	0.41229 (10)	0.7205 (2)	0.82676 (6)	0.0229 (3)	
C11	0.38360 (12)	0.5284 (2)	0.80795 (7)	0.0312 (3)	
H11	0.3807	0.4280	0.8402	0.037*	
C12	0.35906 (13)	0.4822 (3)	0.74246 (7)	0.0364 (4)	
H12	0.3390	0.3506	0.7301	0.044*	
C13	0.36370 (13)	0.6264 (3)	0.69533 (7)	0.0369 (4)	
H13	0.3467	0.5943	0.6505	0.044*	
C14	0.39293 (13)	0.8170 (3)	0.71325 (7)	0.0361 (4)	
H14	0.3968	0.9162	0.6807	0.043*	
C15	0.41687 (12)	0.8646 (2)	0.77900 (6)	0.0297 (3)	
H15	0.4364	0.9967	0.7912	0.036*	
C16	0.43710 (11)	0.9796 (2)	0.91178 (6)	0.0257 (3)	
C17	0.56995 (11)	0.7062 (2)	0.91202 (6)	0.0255 (3)	
H17A	0.5963	0.7450	0.9572	0.031*	
H17B	0.5765	0.5603	0.9086	0.031*	
C18	0.70813 (15)	1.0295 (3)	0.81948 (8)	0.0472 (5)	
H18	0.7352	1.1567	0.8096	0.057*	
C19	0.66923 (13)	0.7306 (3)	0.81087 (7)	0.0357 (4)	
H19	0.6603	0.5979	0.7958	0.043*	

### Atomic displacement parameters (Å^2^)

**Table:**

	*U*^11^	*U*^22^	*U*^33^	*U*^12^	*U*^13^	*U*^23^
Cl1	0.0454 (3)	0.0820 (4)	0.0822 (4)	−0.0076 (3)	0.0220 (2)	0.0428 (3)
N1	0.0555 (8)	0.0248 (8)	0.0306 (7)	0.0036 (7)	0.0081 (6)	0.0001 (6)
N2	0.0283 (6)	0.0304 (7)	0.0253 (6)	−0.0013 (5)	0.0069 (5)	0.0004 (5)
N3	0.0489 (8)	0.0351 (8)	0.0384 (7)	−0.0131 (7)	0.0195 (6)	−0.0055 (6)
N4	0.0514 (8)	0.0463 (10)	0.0397 (7)	−0.0003 (7)	0.0240 (6)	0.0004 (7)
C1	0.0448 (9)	0.0389 (10)	0.0317 (8)	−0.0065 (8)	0.0057 (6)	−0.0035 (7)
C2	0.0441 (9)	0.0366 (10)	0.0529 (10)	−0.0123 (8)	0.0020 (8)	0.0032 (8)
C3	0.0275 (7)	0.0508 (12)	0.0455 (9)	−0.0038 (8)	0.0086 (6)	0.0185 (8)
C4	0.0521 (10)	0.0585 (13)	0.0388 (9)	−0.0014 (10)	0.0241 (8)	0.0008 (9)
C5	0.0474 (9)	0.0360 (10)	0.0382 (8)	−0.0064 (8)	0.0164 (7)	−0.0052 (7)
C6	0.0231 (6)	0.0334 (9)	0.0271 (7)	0.0002 (6)	0.0036 (5)	0.0036 (6)
C7	0.0317 (7)	0.0344 (9)	0.0297 (7)	0.0026 (7)	0.0084 (6)	0.0068 (7)
C8	0.0299 (7)	0.0226 (8)	0.0193 (6)	−0.0001 (6)	0.0054 (5)	0.0023 (6)
C9	0.0274 (6)	0.0185 (7)	0.0200 (6)	0.0006 (6)	0.0053 (5)	0.0021 (5)
C10	0.0243 (6)	0.0246 (8)	0.0202 (6)	0.0026 (6)	0.0046 (5)	0.0004 (6)
C11	0.0427 (8)	0.0269 (9)	0.0245 (7)	0.0000 (7)	0.0055 (6)	0.0024 (6)
C12	0.0482 (9)	0.0306 (9)	0.0302 (8)	0.0004 (8)	0.0024 (6)	−0.0069 (7)
C13	0.0427 (8)	0.0468 (11)	0.0208 (7)	0.0091 (8)	0.0014 (6)	−0.0042 (7)
C14	0.0465 (9)	0.0397 (10)	0.0223 (7)	0.0063 (8)	0.0045 (6)	0.0075 (7)
C15	0.0385 (8)	0.0267 (9)	0.0242 (7)	0.0014 (7)	0.0036 (6)	0.0038 (6)
C16	0.0334 (7)	0.0257 (9)	0.0186 (6)	−0.0012 (7)	0.0053 (5)	0.0032 (6)
C17	0.0288 (7)	0.0262 (8)	0.0220 (6)	0.0010 (6)	0.0048 (5)	0.0036 (6)
C18	0.0574 (11)	0.0420 (11)	0.0467 (9)	−0.0119 (9)	0.0284 (8)	−0.0007 (8)
C19	0.0392 (8)	0.0379 (10)	0.0322 (8)	0.0044 (7)	0.0142 (6)	−0.0043 (7)

### Geometric parameters (Å, º)

**Table:**

Cl1—C3	1.7461 (16)	C8—C9	1.5492 (18)
N1—C16	1.1445 (19)	C8—H8A	0.9900
N2—C19	1.3376 (18)	C8—H8B	0.9900
N2—N3	1.3585 (18)	C9—C16	1.479 (2)
N2—C17	1.4444 (17)	C9—C10	1.5332 (17)
N3—C18	1.3178 (19)	C9—C17	1.5537 (18)
N4—C19	1.314 (2)	C10—C15	1.386 (2)
N4—C18	1.351 (2)	C10—C11	1.388 (2)
C1—C6	1.379 (2)	C11—C12	1.386 (2)
C1—C2	1.382 (2)	C11—H11	0.9500
C1—H1	0.9500	C12—C13	1.377 (2)
C2—C3	1.372 (2)	C12—H12	0.9500
C2—H2	0.9500	C13—C14	1.376 (2)
C3—C4	1.367 (3)	C13—H13	0.9500
C4—C5	1.388 (2)	C14—C15	1.392 (2)
C4—H4	0.9500	C14—H14	0.9500
C5—C6	1.382 (2)	C15—H15	0.9500
C5—H5	0.9500	C17—H17A	0.9900
C6—C7	1.5124 (19)	C17—H17B	0.9900
C7—C8	1.5273 (19)	C18—H18	0.9500
C7—H7A	0.9900	C19—H19	0.9500
C7—H7B	0.9900		
			
C19—N2—N3	109.39 (12)	C16—C9—C8	106.43 (11)
C19—N2—C17	130.06 (14)	C10—C9—C8	114.28 (11)
N3—N2—C17	119.81 (12)	C16—C9—C17	109.49 (12)
C18—N3—N2	101.96 (13)	C10—C9—C17	108.56 (10)
C19—N4—C18	102.36 (13)	C8—C9—C17	107.94 (10)
C6—C1—C2	121.52 (15)	C15—C10—C11	118.93 (13)
C6—C1—H1	119.2	C15—C10—C9	120.87 (13)
C2—C1—H1	119.2	C11—C10—C9	119.90 (12)
C3—C2—C1	118.87 (17)	C12—C11—C10	120.47 (14)
C3—C2—H2	120.6	C12—C11—H11	119.8
C1—C2—H2	120.6	C10—C11—H11	119.8
C4—C3—C2	121.31 (15)	C13—C12—C11	120.22 (16)
C4—C3—Cl1	119.53 (14)	C13—C12—H12	119.9
C2—C3—Cl1	119.16 (15)	C11—C12—H12	119.9
C3—C4—C5	119.02 (16)	C14—C13—C12	119.92 (14)
C3—C4—H4	120.5	C14—C13—H13	120.0
C5—C4—H4	120.5	C12—C13—H13	120.0
C6—C5—C4	121.15 (17)	C13—C14—C15	120.11 (14)
C6—C5—H5	119.4	C13—C14—H14	119.9
C4—C5—H5	119.4	C15—C14—H14	119.9
C1—C6—C5	118.10 (14)	C10—C15—C14	120.35 (15)
C1—C6—C7	120.59 (13)	C10—C15—H15	119.8
C5—C6—C7	121.30 (15)	C14—C15—H15	119.8
C6—C7—C8	111.85 (12)	N1—C16—C9	175.67 (15)
C6—C7—H7A	109.2	N2—C17—C9	112.33 (11)
C8—C7—H7A	109.2	N2—C17—H17A	109.1
C6—C7—H7B	109.2	C9—C17—H17A	109.1
C8—C7—H7B	109.2	N2—C17—H17B	109.1
H7A—C7—H7B	107.9	C9—C17—H17B	109.1
C7—C8—C9	114.21 (11)	H17A—C17—H17B	107.9
C7—C8—H8A	108.7	N3—C18—N4	115.46 (16)
C9—C8—H8A	108.7	N3—C18—H18	122.3
C7—C8—H8B	108.7	N4—C18—H18	122.3
C9—C8—H8B	108.7	N4—C19—N2	110.83 (15)
H8A—C8—H8B	107.6	N4—C19—H19	124.6
C16—C9—C10	110.05 (11)	N2—C19—H19	124.6
			
C19—N2—N3—C18	−0.44 (17)	C16—C9—C10—C11	−159.29 (13)
C17—N2—N3—C18	−171.49 (14)	C8—C9—C10—C11	−39.62 (17)
C6—C1—C2—C3	−1.1 (3)	C17—C9—C10—C11	80.90 (15)
C1—C2—C3—C4	−0.2 (3)	C15—C10—C11—C12	−0.5 (2)
C1—C2—C3—Cl1	179.73 (13)	C9—C10—C11—C12	−174.29 (13)
C2—C3—C4—C5	1.0 (3)	C10—C11—C12—C13	0.5 (2)
Cl1—C3—C4—C5	−178.99 (14)	C11—C12—C13—C14	0.1 (2)
C3—C4—C5—C6	−0.4 (3)	C12—C13—C14—C15	−0.6 (2)
C2—C1—C6—C5	1.5 (2)	C11—C10—C15—C14	0.0 (2)
C2—C1—C6—C7	−179.47 (15)	C9—C10—C15—C14	173.75 (13)
C4—C5—C6—C1	−0.8 (2)	C13—C14—C15—C10	0.5 (2)
C4—C5—C6—C7	−179.76 (15)	C19—N2—C17—C9	−94.71 (18)
C1—C6—C7—C8	−77.25 (17)	N3—N2—C17—C9	74.23 (16)
C5—C6—C7—C8	101.70 (17)	C16—C9—C17—N2	−65.98 (14)
C6—C7—C8—C9	174.47 (12)	C10—C9—C17—N2	54.17 (16)
C7—C8—C9—C16	60.28 (15)	C8—C9—C17—N2	178.56 (11)
C7—C8—C9—C10	−61.39 (16)	N2—N3—C18—N4	0.2 (2)
C7—C8—C9—C17	177.73 (12)	C19—N4—C18—N3	0.0 (2)
C16—C9—C10—C15	27.07 (17)	C18—N4—C19—N2	−0.34 (19)
C8—C9—C10—C15	146.73 (13)	N3—N2—C19—N4	0.52 (18)
C17—C9—C10—C15	−92.74 (15)	C17—N2—C19—N4	170.36 (14)

### Hydrogen-bond geometry (Å, º)

**Table:**

*D*—H···*A*	*D*—H	H···*A*	*D*···*A*	*D*—H···*A*
C8—H8*A*···N1^i^	0.99	2.53	3.522 (2)	178
C11—H11···N1^i^	0.95	2.60	3.533 (2)	166
C17—H17*A*···N1^ii^	0.99	2.58	3.5101 (18)	156
C18—H18···N4^iii^	0.95	2.46	3.277 (2)	144

Symmetry codes: (i) *x*, *y*−1, *z*; (ii) −*x*+1, −*y*+2, −*z*+2; (iii) −*x*+3/2, *y*+1/2, −*z*+3/2.

## References

[bb1] Brandenburg, K. (2010). *DIAMOND*. Crystal Impact GbR, Bonn, Germany.

[bb2] Bruker (2013). *APEX2*, *SAINT* and *SADABS*. Bruker AXS Inc., Madison, Wisconsin, USA.

[bb3] Li, Y., Dong, F., Liu, X., Xu, J., Li, J., Kong, Z., Chen, X. & Zheng, Y. (2012). *Environ. Sci. Technol.* **46**, 2675–2683. 10.1021/es203320x 22339258

[bb4] Rizzoli, C., Marku, E. & Greci, L. (2009). *Acta Cryst.* E**65**, o663. 10.1107/S1600536809007120 PMC2969042 21582408

[bb5] Sheldrick, G. M. (2008). *Acta Cryst.* A**64**, 112–122. 10.1107/S0108767307043930 18156677

[bb6] Sheldrick, G. M. (2015). *Acta Cryst.* C**71**, 3–8.

[bb7] Yin, B.-T., Yan, C.-Y., Peng, X.-M., Zhang, S.-L., Rasheed, S., Geng, R.-X. & Zhou, C.-H. (2014). *Eur. J. Med. Chem.* **71**, 148–159. 10.1016/j.ejmech.2013.11.003 24291568

