# Supplementary figures and images for: Crystal structure of fenbuconazole
Source: Acta Crystallogr E Crystallogr Commun. 2015 Aug 22;71(Pt 9):o680–1. doi: 10.1107/S205698901501542X (PMC4555419; doi:10.1107/S205698901501542X)

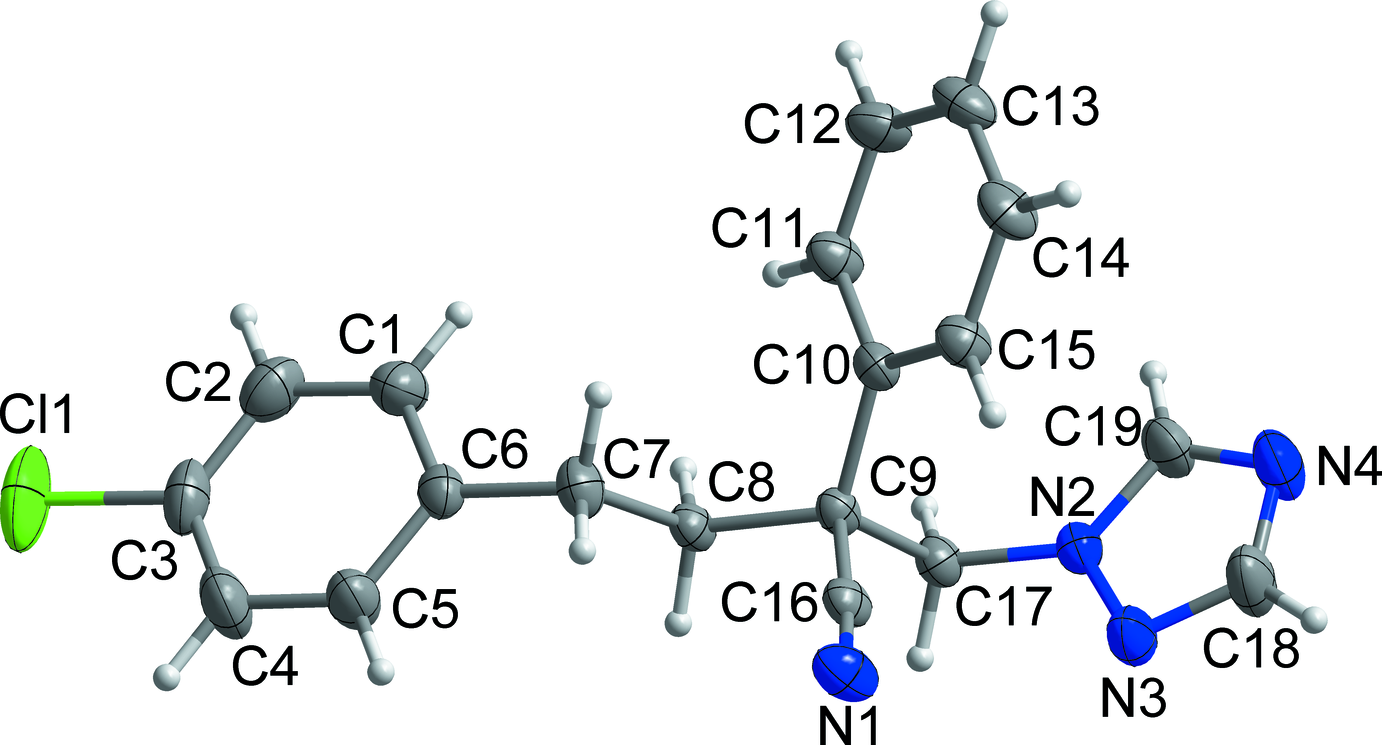

Supplement: Supplementary file 4 [file e-71-0o680-fig1.tif]

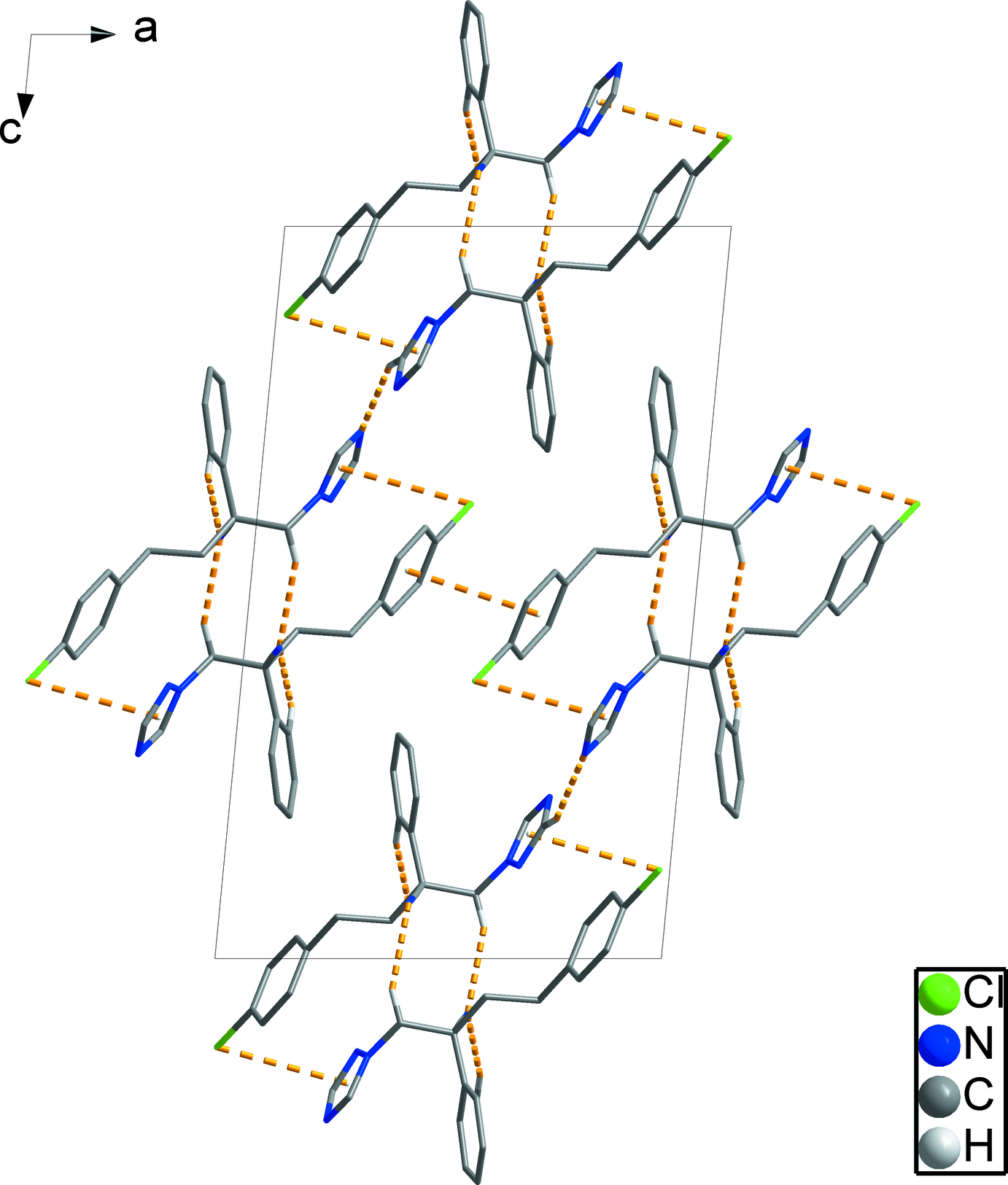

Supplement: Supplementary file 5 [file e-71-0o680-fig2.tif]
